# The Importance of Cleanliness

**Published:** 1887-01

**Authors:** 


### The Importance of Cleanliness.

In the annual discourse before the Massachusetts Medical Society at its last annual meeting, Dr. R. M. Hodges says:

"Dirty finger-nails may communicate a fatal poison, through the trivial operations of surgery which every physician undertakes to perform; or inaugurate the 'private pestilence' which still sometimes follows in the track of the obstetrician."

